# Biocompatibility of artificial bone based on vancomycin loaded mesoporous silica nanoparticles and calcium sulfate composites

**DOI:** 10.1007/s10856-016-5671-z

**Published:** 2016-02-16

**Authors:** Jisheng Gu, Teng Wang, Guoxin Fan, Junhua Ma, Wei Hu, Xiaobing Cai

**Affiliations:** Department of Orthopedics, Shanghai Tenth People’s Hospital, Tongji University School of Medicine, Yanchang Road 301, Shanghai, 200072 China; The First Clinical Medical College of Nanjing Medical University, Nanjing, Jiangsu 210029 China; Department of General Surgery, Changhai Hospital, Second Military Medical University, Changhai Road 168, Shanghai, 200433 China

## Abstract

The aim of this study was to evaluate the in vitro and in vivo biocompatibility of artificial bone based on vancomycin loaded mesoporous silica nanoparticles and calcium sulfate composites. In vitro cytotoxicity tests by cholecystokinin octapeptide (CCK-8) assay showed that the 5 %Van-MSN-CaSO_4_ and Van-CaSO_4_ bone cements were cytocompatible for mouse osteoblastic cell line MC3T3-E1. The microscopic observation confirmed that MC3T3-E1cells incubated with Van-CaSO_4_ group and 5 %Van-MSN-CaSO_4_ group exhibited clear spindle-shaped changes, volume increase and maturation, showing that these cements supported adhesion of osteoblastic cells on their surfaces. In addition, the measurement of alkaline phosphatase activity revealed the osteoconductive property of these biomaterials. In order to assess in vivo biocompatibility, synthesized cements were implanted into the distal femur of twelve adult male and female New Zealand rabbits. After implantation in artificial defects of the distal femur, 5 %Van-MSN-CaSO_4_ and Van-CaSO_4_ bone cements did not damage the function of main organs of rabbits. In addition, the Van-MSN-CaSO_4_ composite allowed complete repair of bone defects with new bone formation 3 months after implantation. These results show potential application of Van-MSN-CaSO_4_ composites as bone graft materials for the treatment of open fracture in human due to its mechanical, osteoconductive and potential sustained drug release characteristics and the absence of adverse effects on the body.

## Introduction

Bone transplants are broadly used in the treatment of open fractures, especially for providing mechanical or structural support and promoting the bone tissue formation [1, 2]. Due to high rate of bone infections and infection-related complications in open fractures, it is necessary to load bone implants with antibiotics for treating osteomyelitis or eradicating, reducing or preventing contaminations [3]. In addition, the synthetic bone material must be safe and suitable for implantation in vivo. Calcium sulfate has got a long clinical history as bone biomaterial because apart from providing structural support, it holds good biocompatibility, osteoinductivity and bioabsorbability and can serve as a vehicle for delivery and local release of antibiotics [4–8]. For instance, Kelly et al. (2001) successfully used surgical grade calcium sulfate pellets as convenient and safe bone graft substitute for the treatment of patients with bone defects [9]. Additional studies on bone graft substitutes have equally confirmed the biocompatibility of calcium sulfate composites and suggested their use as suitable bone implant material for in vivo therapies [10, 11]. In our previous work, we used calcium sulfate particles impregnated with vancomycin for the treatment of infected bone defects of 28 cases of open fractures among which 26 cases were followed up, and found that this material is beneficial for osteoblasts migration and vascular ingrowth and can successfully promote the fracture healing [12]. However, we recorded some shortcomings, such as low drug delivery and drug release ability and the difficulty to achieve the maintenance of drug concentration. Therefore, a drug delivery material, which can sustain drug release, is needed for the clinical treatment.

In the recent years, the use of mesoporous silica nanoparticles (MSNs) has been extended in biomedical fields such as cancer therapy, drug and gene delivery, biosensors, and enzyme immobilization [13]. MSNs can be used as drug carriers, can sustain delivery and local release of drugs and has good biocompatibility. For example, Kempen et al. [8] showed that mesenchymal stem cells (MSCs) labeled with MSN had no cytotoxicity at the 250 µg/mL concentration required for labeling. It was also reported that multi-functional MSNs coated with poly (N-isopropylacrylamide) (PNIPAM) composite nanomaterials can easily carry guest molecules into human breast carcinoma cells (MCF-7) and display minute cytotoxicity to the cells [14]. Previously, a biocompatible silica-based nanoparticles synthesized by incorporating the bone-forming peptide (BFP) into MSNs showed better cell proliferation and osteogenic differentiation and could be used as a bioactive material for bone repairing, bone regeneration, and bio-implant coating applications [15]. Most of drugs are loaded into the pores of MSN and can only be released by diffusion. This significantly reduces and retards the local drug release and subsequently improves drug bioavailability. However, MSN itself is in a powder state, which makes it difficult to be directly used as bone implant material. Up to now, studies have shown that by using MSNs loading rate of 5 %, artificial bone materials with a relatively good solidity can be obtained [11, 16]. Therefore, by combining MSNs and calcium sulfate, a new type of robust artificial bone material for bone repair with sustained release effect can be obtained. However, to potentiate this material for suitable use in the human body, it is primarily necessary to investigate its impacts on the organisms.

Therefore, in the present study, we aimed to examine the biocompatibility of the previously synthesized artificial bone based on vancomycin loaded mesoporous silica nanoparticle and calcium sulfate composites [17] in order to provide experimental basis for its application as a bone graft materials.

## Materials and methods

### In vitro biocompatibility evaluation of bone cements

#### Materials

The cells of murine osteoblast MC3T3-E1 were purchased from the Cell Bank of the Chinese Academy of Sciences (Shanghai, China). Penicillin, streptomycin, fetal bovine serum (FBS) and α-MEM medium were all purchased from Hyclone (Logan, UT). The artificial bone based on vancomycin loaded MSNs and calcium sulfate composites was obtained from the Key Laboratory of Molecular Engineering of Polymers of Fudan University. The medical grade calcium sulfate was purchased from Tianjin Sheehan biochemical technology co., LTD. Cholecystokinin octapeptide (CCK-8) was purchased from Peptide Institute (Minoh, Japan) while alkaline phosphatase (ALP) detection kit was provided by Nanjing Jiancheng Bioengineering Institute.

#### Preparation of leaching solutions

Bone cement based on vancomycin loaded MSNs and calcium sulfate composites (5 %Van-MSN-CaSO_4_) [11] and calcium sulfate impregnated with vancomycin (Van-CaSO_4_) bone implant [12] were prepared as described elsewhere [11, 12]. A certain amount of prepared Van-MSN-CaSO_4_ or Van-CaSO_4_ was sterilized under ultraviolet radiation, soaked in α-MEM medium supplemented with 10 % FBS at a soaking rate of 0.1 g/ml according to ISO10993.5 standards and incubated hermetically at 37 °C for 72 h. Subsequently, the obtained leaching solutions were filtered and stored at 4 °C until use.

#### Cell cultivation, cytotoxicity evaluation and alkaline phosphatase activity

MC3T3-E1 cells were cultured in α-MEM culture medium supplemented with 10 % (v/v) FBS (Hyclone, USA). Incubation was performed in a CO_2_ incubator (5 % CO_2_, 95 % air). The cells were subcultured every 2 or 3 days.

To evaluate the cytotoxicity of artificial bone composites, third-generation MC3T3-E1 cells were seeded in 96-well plates (4000 cells/well containing 100 µl of α-MEM medium in 5 % CO_2_ controlled air incubator at 37 °C for 24 h to allow cell attachment. After cell adhesion on the bottom of the wells, the culture medium was aspirated and changed into fresh medium supplemented with dilutions of the leaching solutions (100, 50 and 25 % concentrations of filtrated leaching solutions) prepared as indicated in Table 1. Six wells were seeded for each dilution and the negative control for which no leaching solution was added. After 1, 2, 3 and 5 days cultivation, cells were washed twice with fresh α-MEM medium and replaced by 100 µl of the same medium plus 10 µl of CCK-8. After further cultivation for 2 h, we measured the absorbance at 450 nm using the ELISA plate reader according to the guidelines of CCK8 assay kit.

**Table 1:** Experimental groups and interventions

Experimental group	Implementation plan
A (100 %Van-MSN-CaSO_4_ original leaching solution)	3 ml Van-MSN-CaSO_4_ original solution
B (50 %Van-MSN-CaSO_4_ dilution)	1.5 ml A + 1.5 ml α-MEM medium supplemented with 10 % FBS
C (25 % Van-MSN-CaSO_4_ dilution)	0.5 ml A + 1.5 ml α-MEM medium supplemented with 10 % FBS
D (100 %Van-CaSO_4_ original leaching solution)	3 ml Van-CaSO_4_ original solution
E (50 %Van-CaSO_4_ dilution)	1.5 ml D + 1.5 ml α-MEM medium supplemented with 10 % FBS
F (25 %Van-CaSO_4_ dilution)	0.5 ml D + 1.5 ml α-MEM medium supplemented with 10 % FBS
G(negative control)	3 ml α-MEM medium supplemented with 10 % FBS

For ALP assay, the cells were cultured under the same conditions as that of the CCK8 assay. After 3 and 5 days, ALP activity was measured by spectrophotometric methods at 520 nm using an ALP assay kit according to the manufacturer’s protocol.

### In vivo biocompatibility evaluation of bone cements

#### Animal population

Twelve adult male and female New Zealand rabbits, with a mean weight of 2.5 kg and ages ranging from 4 to 5 months were divided into control group (n = 3), simple MSNs implant group (n = 3), 5 %Van-MSN-CaSO_4_ implant group (n = 3) and CaSO_4_ implant group (n = 3). All procedures were performed in accordance with standard guidelines as described in the Guide for the Care and Use of Laboratory Animals (US National Institutes of Health 85-23, revised 1996). All animal protocols were agreed by the local Institutional Animal Care and Use Committee of Tongji University.

#### Surgical procedure

**Fig. 1:**
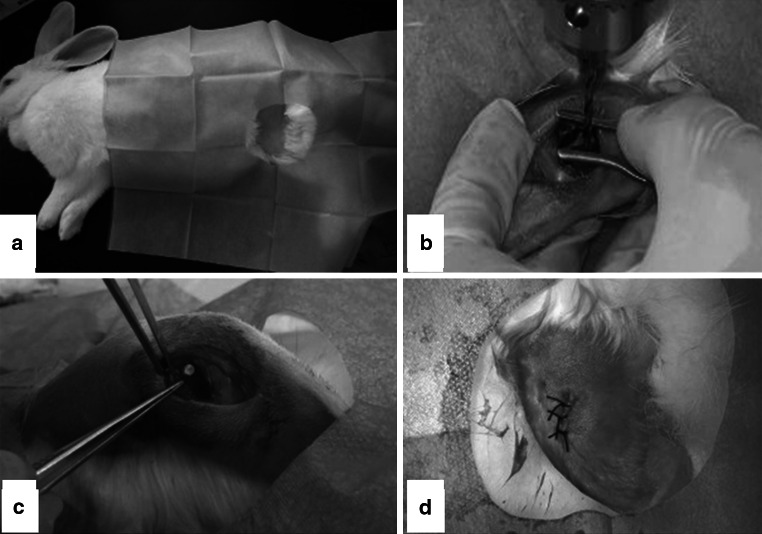
Images of the surgical procedures adopted for implantation of artificial bones. **a** Shaving and desinfection, **b** perforation to create bone defect, **c** implantation of artificial bones in created defects, **d** sutures

The surgical procedure adopted for implantation of bone materials is depicted in Fig. 1. Prior to surgical processes, general anesthesia was achieved by intravenous injections of pentobarbital at a dose of 1 ml/kg body weight whereas 2 % lidocaine was used for local anesthesia in the subcutaneous area of the insertion site. After shaving, povidone iodine disinfection and placing surgical drapes, about 3 cm longitudinal incision was achieved in the area of the distal femoral diaphysis, followed by a step by step separation to expose the lateral condyle cortical bone. Thereafter, bone defects (ø 3 mm, depth 2 mm) were created by drilling perpendicularly to the direction of the cortical bone, with cautious avoidance of the penetration in the lateral cortex. After washing the wound with physiological water, implant materials corresponding to experimental and control groups were inserted. For the blank control group the wound was simply open without implantation of bone materials. Finally, following disinfection, surgical sites were closed using resorbable sutures and rabbits woken up after caging.

#### Samples collection

Blood samples were taken respectively at 1, 2 and 3 months after operation, centrifuged at 3000 g for 5 min for serum separation and cryopreserved at −80 °C.

For collection of bone samples and main viscera, animals were sacrificed after 12 weeks by intravenous injection of 20 ml of air. Distal femurs of rabbits containing defects and major rabbit organs (heart, liver, spleen, lung, and kidney) were harvested en bloc after operation and immersed in paraformaldehyde until further processing for histological analysis. In histological analysis of viscera, samples were collected after sacrifice of normal animals without implants or defects.

#### Histological and histopathological evaluation

The conventional HE staining was used for microscopic examination of paraffin embedded histological sections of main organs (5–6 microns thickness). Bone specimens were decalcified and HE stained. The decalcified sections were observed under a light microscope (Nikon Eclipse 50i) with a 10x magnification objective. Eight nonconsecutive images of each HE-stained specimen section were blindly analyzed by one expert observer. The images were digitally captured (Nikon H500S) and visualized with Image Pro-Plus 6.0 for Windows.

#### Micro-CT examination

The micro-CT analysis was performed on samples from the lateral femur condyle (including the defect with implant material) using the high-resolution micro-CT system Skyscan 1076 (SkyScan, Aartselaar, Belgium). The X-ray source was an air-cooled, sealed Hamamatsu 100/250 tube with a focal spot size <8 µm. The X-ray tube was run at source voltage of 40 kV and source current of 250 µA without filter. The X-ray detector consisted of a Princeton Instruments camera with camera pixel size of 12.60 µm and camera X/Y Ratio of 1.0000. NRecon program version 1.6.9.4 was employed for reconstruction.

The quality of new bone was evaluated based on pictures analyzed by CTAn, a micro-CT analysis program. The bony tissue of the new bone was analyzed with a ROI (region of interest) at a width of 0.99 mm and a height of 2.48 mm. The bone portion containing implants (defects zone) was analyzed using an ROI at a size of 0.97 mm and a height of 2.48 mm. For quantification of the quantity and quality of the new bone, we evaluated the percent bone volume (BV/TV) and specific bone surface (BS/BV) as well as trabecular thickness (Tb·Th) and trabecular number (Tb·N).

#### Statistical analysis

The experimental data were expressed as mean ± standard deviation. SPSS20.0 statistical software was used for statistical analysis using one way-ANOVA for comparison between multiple samples. Multiple intra-groups comparisons were achieved through SNK test. Statistical significance was evaluated with a p-values cutoff of <0.05.

## Results

### In vitro biocompatibility evaluation of bone cements

#### Cytotoxicity test

**Fig. 2:**
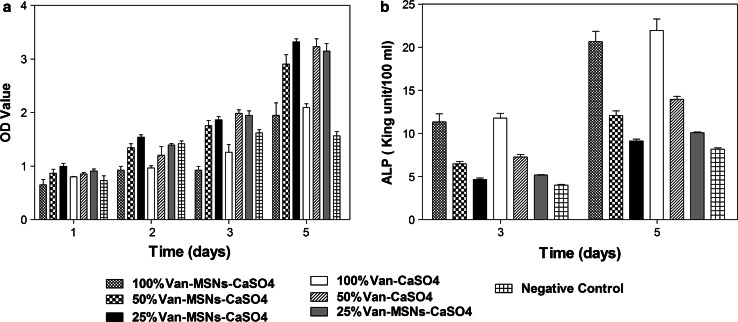
*In vitro* analysis of the biocompatibility of different artificial bone leaching solutions measured after 5 days cultivation: **a** Proliferation of MC3T3-E1 cells, **b** phosphatase alkaline (ALP) activity of MC3T3-E1 cells

In order to evaluate the cyto-compatibility of bone implant materials, the CCK-8 cytotoxicity assay was performed after 1, 2, 3 and 5 days cultivation of MC3T3-E1 cells with different concentrations of leaching solutions stemmed from bone implants. As displayed in Fig. 2a, an increase in absorbance from day 1 to day 7 was recorded, which indicates cell viability with both the Van-CaSO_4_ and Van-MSN-CaSO_4_ implant materials. Moreover, the proliferation behaviors of cells with different implant materials were very similar to each other in every measurement point. Indeed, comparatively to the negative control group and the Van-CaSO_4_ artificial bone control group, Van-MSN-CaSO_4_ implant material had no inhibitory effect on the proliferation of mice osteoblasts (P > 0.01) and there was no significant difference between groups or between concentration groups (*P* > 0.01).

#### Alkaline phosphatase assay

To assess the effect of bone cements on osteoblast differentiation, we determined the ALP activity of MC3T3-E cells after 3 and 5 days cultivation. The measurement of ALP activity (Fig. 2b) showed significant differences between the blank control group and the leaching solutions with dilution rates ≥50 %. Between leaching solutions with dilution rate of 25 % and the blank control, no statistically significant differences were observed. No significant differences were observed among Van-MSN-CaSO_4_ and Van-CaSO_4_ leaching solutions. Nevertheless, MC3T3-E1 cells produced increasing ALP activity with the increase of the concentrations of leaching solutions. This result indicated the osteoconductivity of both kinds of bone cements which must be attributed to Ca^2+^ ions in a dose-dependent way. We could additionally notice an increase of ALP activity with the incubation time. The findings similarly revealed improved differentiation of MC3T3-E1cells seeded in the presence of different concentrations of Van-MSN-CaSO_4_ or Van-CaSO_4._

#### Effect of bone cements on cytological changes

**Fig. 3:**
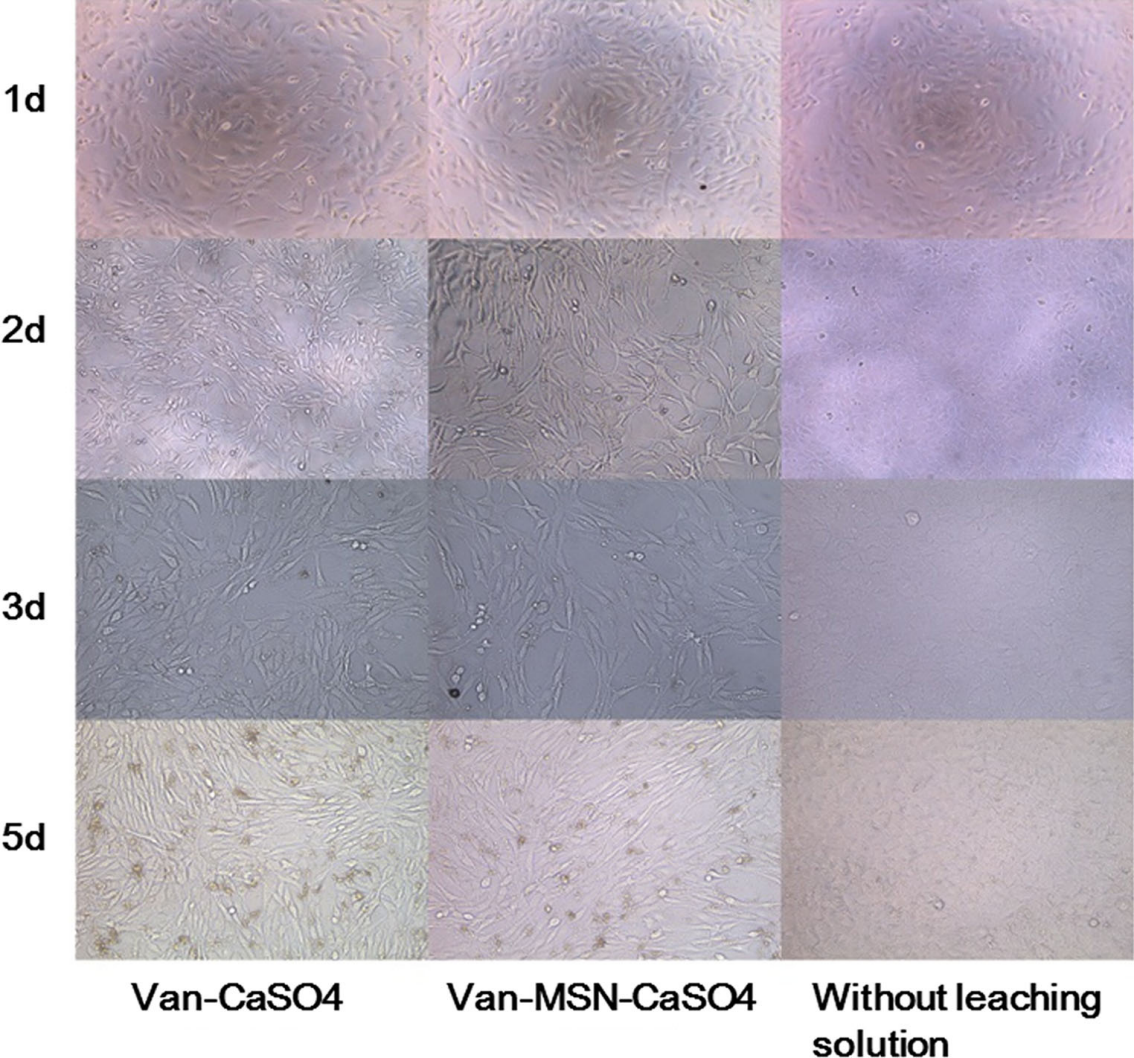
Cytological analysis of morphological changes of MC3T3-E1 cells after incubation with different bone cements

The cytological examination of MC3T3-E1 osteoblasts incubated with media containing prepared leaching solutions is depicted on Fig. 3. The microscopic observation of cell morphological changes revealed that cells incubated with Van-CaSO_4_ and 5 %Van-MSN-CaSO_4_ leaching solutions were mature, clearly fusiform with increased volume. Cells in the control group without leaching solution remained polygonal and did not show obvious cell differentiation. This suggested that studied bone cements supported adhesion of osteoblastic cells on their surfaces. Altogether, the results of the in vitro biocompatibility assessment showed that the Van-MSN-CaSO_4_ implant presents no obvious cytotoxicity and could be used for clinical applications.

### In vivo evaluation of the biocompatibility of bone cements

#### Macroscopic observations

**Fig. 4:**
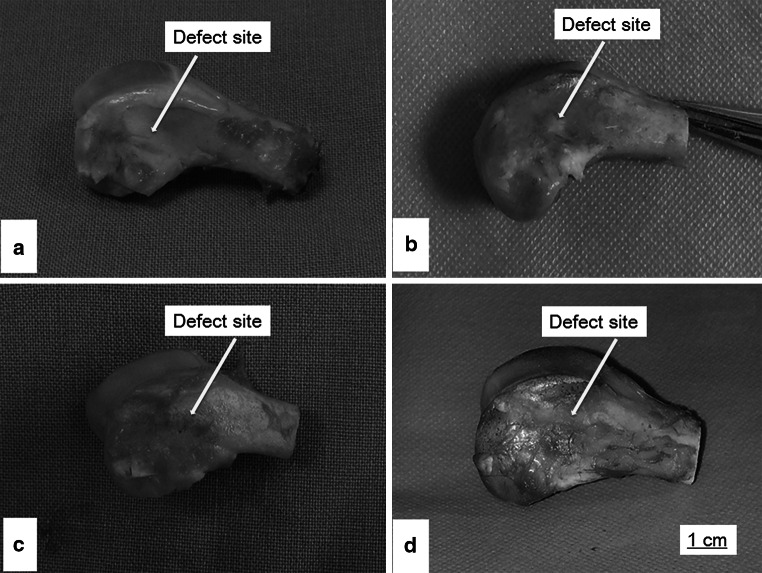
Images depicting the macroscopic state of bony defects 3 months after implantation. **a** pure MSN (SiO2) implant group, **b** van-CaSO_4_ implant group, **c** van-MSN-CaSO_4_ implant group, **d** pure control group of rabbits without implant (Group 4)

In order to evaluate the efficacy of Van-CaSO_4_ and 5 %Van-MSN-CaSO_4_ implants in defects repair, animals were killed 3 months after bone implantation. After surgery lower extremities of femur containing defects were collected and macroscopically examined. The photographs are presented in Fig. 4. The results showed that in the MSN implant group, most of the bone defects was filled with SiO_2_ granules. No bony connection was seen in any of the 3 rabbits, but partial bone formation was observed (Fig. 4a). In Van-CaSO_4_ group, most of the defect was healed and only slight bone formation was found in sites of the defects (Fig. 4b). In the Van-CaSO_4_-MSN group, complete healing of the segmental defect was observed in all 3 rabbits. In addition remarkable new bone formation (Fig. 4c) and no residual CaSO_4_ was found in the 3 rabbits. In the control group without implant, no new bone formation was found in sites of the defects (Fig. 4d).

#### Micro-CT analysis

**Fig. 5:**
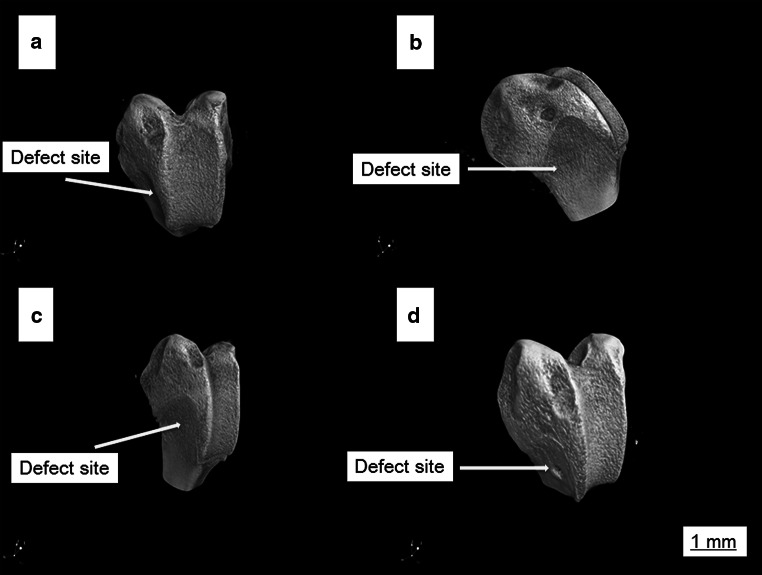
Micro-CT images of femur regions containing bony defects 3 months after implantation. **a** Pure MSN (SiO2) implant group, **b** van-CaSO_4_ implant group, **c** van-MSN-CaSO_4_ implant group, **d** pure control group of rabbits without implant (Group 4)

**Fig. 6:**
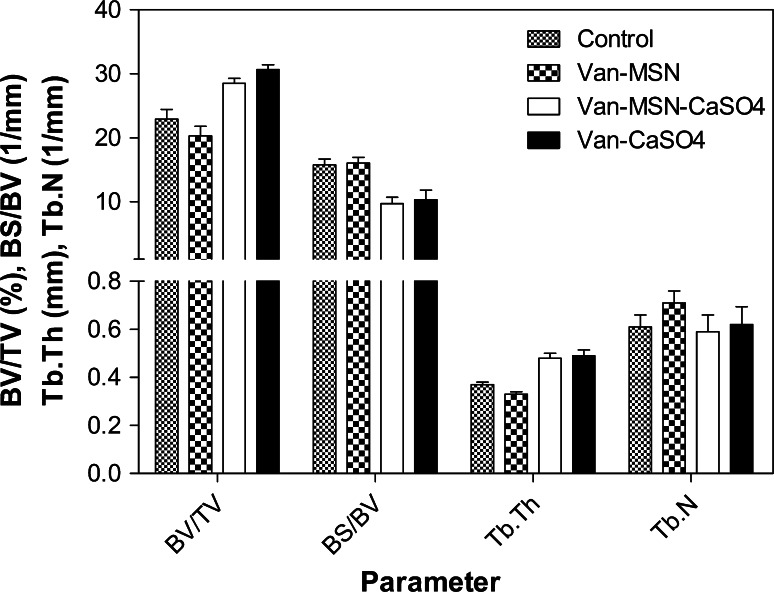
Descriptive statistics for BV/TV, BS/BV, Tb·Th and Tb·N measured by micro-CT

As shown in Fig. 5, the micro-CT analysis of distal femur samples collected 3 months after implantation further confirmed the macroscopic observation results. Descriptive statistics for BV/TV, BS/BV, Tb·Th and Tb.N were determined. The results showed that (Fig. 6), the mean relative BV/TV (newly formed bone volume density) were higher at 12 weeks. The adjusted mean BV/TV were 22.92, 20.30, 28.52 and 30.65 % for control, Van-MSN, Van-MSN-CaSO_4_ and Van-CaSO_4_ implant materials, respectively. The BS/BV value was 15.77 m^−1^ for the control, 16.05 for Van-MSN, 9.69 m^−1^ for Van-MSN-CaSO_4_ and 10.32 m^−1^ for Van-CaSO_4_. The differences of BV/TV and BS/BV between Van-MSN-CaSO_4_ and Van-CaSO_4_ implant materials were not statistically significant. The trabecular thickness (Tb·Th) was smaller in the control and Van-MSN groups compared to implant groups but no statistically significant differences were observed between groups.

#### Histological evaluation

**Fig. 7:**
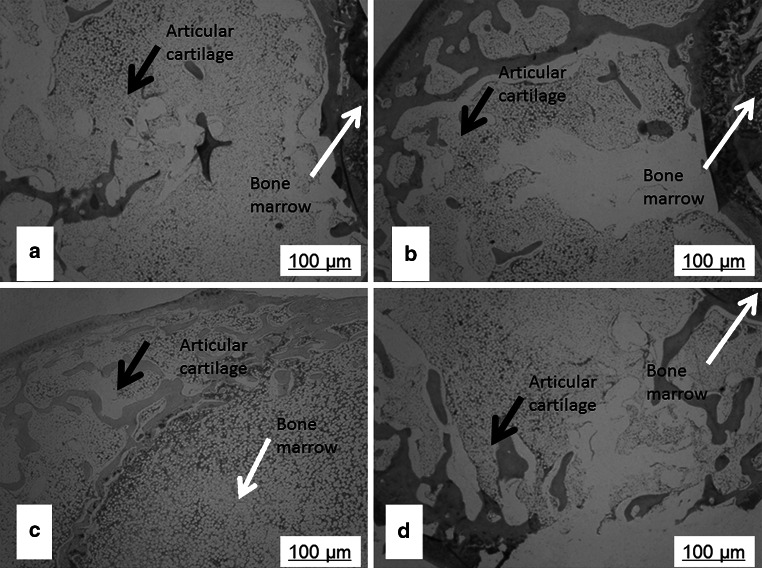
Stained histology of femur regions containing bony defects 3 months after implantation. **a** Pure MSN (SiO2) implant group, **b** van-CaSO_4_ implant group, **c** van-MSN-CaSO_4_ implant group, **d** pure control group of rabbits without implant (Group 4)

The HE staining and histological observation of decalcified bones including the defect site was performed 12 weeks after implantation. The results (Fig. 7) revealed that in the rabbits femur bone defects, distinctive lamellar bone developed at the host bone-implant boundary and within the spongy spaces of the Van-MSN-CaSO_4_ implants (Fig. 7b), as well as in the Van-CaSO_4_ implants (Fig. 7c). Small amount of new bone were similarly formed in Van-MSN implants (SiO4 alone) (Fig. 7a) while only loose connective tissue were noticed in the gap control group (Fig. 7d).

#### Pathomorphological observation of viscera

**Fig. 8:**
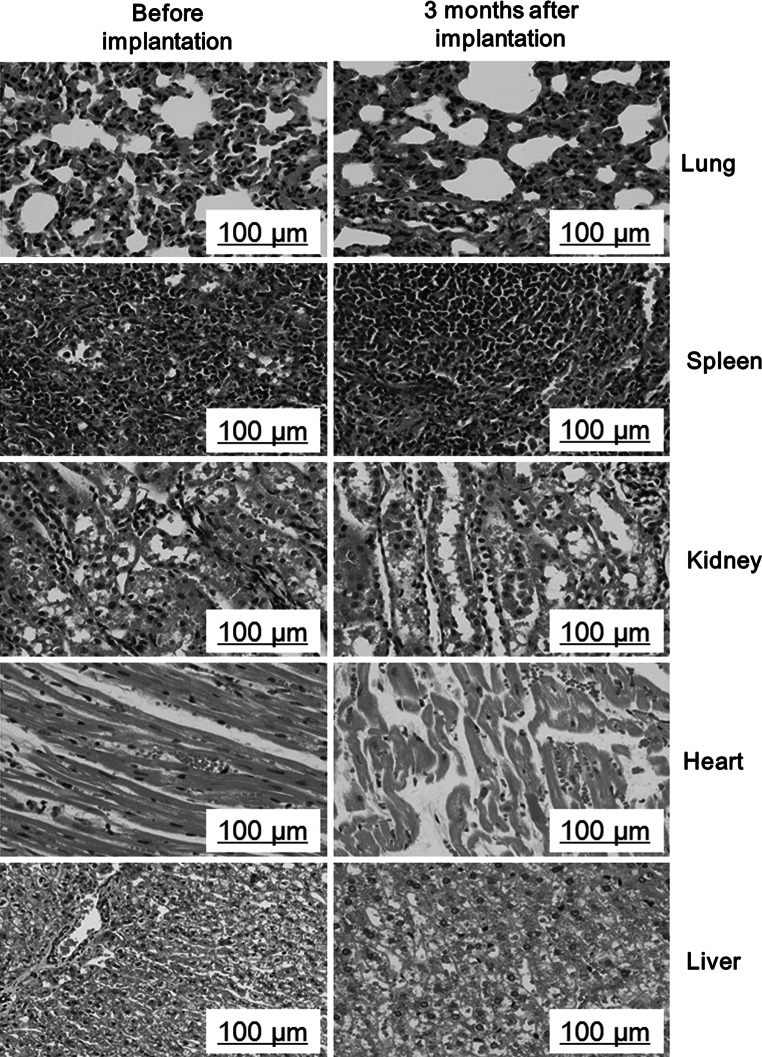
Histopathological analysis of lung, spleen, kidney, heart and liver before and 3 months after implantation of Van-MSN-CaSO_4_ implant

In order to evaluate the toxicity of implants material, HE staining and histopathological observation of sections of main organs before and after implantation of Van-MSN-CaSO_4_ were performed. The result (Fig. 8) showed no visible significant differences of the effect of different implant groups on heart, liver, spleen, lung, kidney and other important organs. This suggested that Van-MSN-CaSO_4_ implant is biocompatible and can be used as a safe bone implant for treatment of bony defects.

#### Hematological index

In order to further understand the cytotoxicity of artificial bone composite from biochemical point of view, we additionally determined the hematological index of studied rabbits. As shown in Table 2, no statistically significant difference was observed between groups, which further confirms the biocompatibility of Van-MSN-CaSO_4._

**Table 2:** Serum biochemical indexes of each rabbit measured before and after implantation of bone composites. G1: group1 (pure MSN (SiO2) implant group), G2: Group 2 (Van-CaSO_4_-MSN (SiO2–CaSO_4_) implant group) G3: Group 3 (CaSO_4_ implant group), G4: Group 4 (the control group without implant), N1–N12: rabbit numbers

Sample number	ALT	AST	GGTLI(r-GT)	UREA	CREA	ALP
G1N1	30.1	26.7	10.2	11.7	68.5	68.7
G1N2	62.6	20.2	12.3	8.8	73.2	46.4
G1N3	60.5	15.1	12.3	10.6	81.7	49.4
G2N4	33	22.2	8.2	7.3	70.1	57.5
G2N5	59.8	25	9.5	11.5	74.4	57.6
G2N6	45.4	23.5	7.5	10.3	65.3	87.1
G3N7	28.7	17.7	8.3	12.5	66.4	44.3
G3N8	41.9	21.2	8.5	12.2	83.2	90.5
G3N9	65.9	23.2	6.1	6.9	77.6	42
G4N10	61.4	35.2	11.5	11.3	54.7	63.3
G4N11	41.9	20.7	10.6	12.5	98.9	30.7
G4N12	77.8	30.9	9.6	7.1	103.8	31.6
Before operation	22.2	14	8.1	6.3	52.8	63
G2-2 days after operation	33.5	56.4	8	9.3	58.6	33.4

## Discussion

Good biocompatibility is an imperative parameter to take into account during the development of bone implant materials. In the present study, the in vitro and in vivo evaluation of Van-MSN-CaSO_4_ and Van-CaSO_4_ biocompatibility was performed in order to assess their safety and potential usefulness in bone transplantation.

The cell proliferation experiment revealed that there was no significant difference of the absorbance of MC3T3-E1 cells incubated with leaching solution originated from Van-MSN-CaSO_4_ composite when compared with Van-CaSO_4_ control group (*P* < 0.05). The optical density (OD) values increased progressively until 5 days cultivation showing that the proliferation activity of MC3T3-E1 cells was not significantly affected. This result implies that Van-MSN-CaSO_4_ composite had no obvious toxic effects on osteoblasts as it was previously reported by Chung et al. [18] who found that MSNs have no significant effect on the proliferation and differentiation of human bone marrow mesenchymal stem cells.

Osteoblasts are main functional cells of bone formation responsible for bone matrix synthesis, secretion and mineralization. As bone implant, MSNs may directly affect the osteogenesis effect of osteoblasts. ALP, as a key indicator of osteoblast maturation and differentiation [19], is of great significance in evaluating the degree of differentiation of osteoblasts. In this study, we found that ALP activity increased significantly (*P* < 0.05) over the incubation time in cultures of MC3T3 cells incubated with 5 %MSNs-CaSO_4_ leaching solution compared with the blank control group. In addition, ALP activity increased obviously with the increase of leaching solution concentration. More importantly, no significant differences were recorded between 5 % MSNs-CaSO_4_ composite leaching solution and the CaSO_4_ leaching solution control group. This further illustrates that corresponding leaching solutions did not negatively affect osteoblast proliferation but promoted cell morphological changes and differentiation. These effects are likely attributable to the Ca^2+^ solution which was previously reported to have osteoconductive effect [19, 20].

The effect of MSNs implants on rabbit’s bone tissue fractures healing process, which is similar to that of human, have not been reported so far. The macroscopic observation and Micro-CT analysis showed that after the implantation of Van-MSN-CaSO_4_ artificial bone composite, complete healing of bony defects was observed 12 weeks after surgery compared with Van-CaSO_4_ composite and the blank control group without implant. This result shows that Van-MSN-CaSO_4_ implant did not delay the healing of bony defects or hinder bone tissue repair, which indicates its potential application as a good drug carrier implant able to concurrently accomplish bone defects filling and local drug release.

After implantation in the distal femur, CaSO_4_ can be absorbed and degraded in the body, vancomycin can enter in the metabolism of liver and kidney but whether SiO2 nanoparticles can produce certain toxicity effect to the body or not is unclear. Therefore, we further investigated the toxicity of bone implant on rabbit internal organs by histopathological observation of major organs. In addition blood biochemical index of the seemingly damaged function of liver and kidney were determined in order to understand the cytotoxicity of artificial bone composites from biochemical point of view. The result showed no significant pathological effect changes in heart, liver, spleen, lung and kidney. According to the blood biochemical test, there was no significant difference between studied groups and Van-MSN-CaSO_4_ material did not cause the body to produce apparent rejective reaction or inflammation. Therefore, the Van-MSN-CaSO_4_ bone implant can be used as the carrier of vancomycin without producing obvious toxic effect. According to previous studies, direct injections of MSNs by peritoneal or intravascular ways do not produce the same effects. In this study, the local implantation of bone materials did not cause much release of MSNs into the bloodstream and could not therefore instigate the damage of main organs.

We employed 5 %Van-MSN-CaSO_4_ rather than using a larger dose of MSNs because previous studies [11, 21] showed that this dose of MSNs is enough to maintain vancomycin concentration and drug release for long time. In addition this concentration is greater than the vancomycin minimal inhibitory concentrations (MIC) of *S. aureus* which is responsible for the common bacterial infection of bone. On the other hand, this proportion of MSNs does not affect the bone filling potential of CaSO_4_ material [12]. One limitation of the present work could be that we did not measure the in vivo release of vancomycin, therefore it would be inaccurate to affirm that the use of MSN allows for sustained drug release. However, this issue was already studied in our previous research work where we thoroughly investigated the drug release efficiency of VAN-MSN-CaSO4 cement [17].

In the process of bone formation, additionally to the osteoblasts, there are many factors that affect bone healing, such as osteoclasts and bone marrow mesenchymal stem cells [22, 23]. We need to further study the toxic effects of MSN materials on these cells, as well as the interaction between silica nanoparticles and organelles. We similarly need to further study safety dose of MSNs after implantation as bone filling material.

## Conclusion

Vancomycin loaded MSNs-CaSO_4_ artificial bone material (MSNs mass fraction of 5 %) showed no obvious cytotoxic effect on MC3T3-E1 cells. The local implantation of this bone implant materials did not delay fracture healing and had no obvious damage effects on the function of main organs of rabbit. In conclusion, Van-MSN-CaSO_4_ artificial bone material has good biocompatibility and could be used for the treatment of bone defects in human since fracture healing in the rabbit is similar to that of human.
